# SigUNet: signal peptide recognition based on semantic segmentation

**DOI:** 10.1186/s12859-019-3245-z

**Published:** 2019-12-20

**Authors:** Jhe-Ming Wu, Yu-Chen Liu, Darby Tien-Hao Chang

**Affiliations:** Department of Electrical Engineering, National Cheng Kung University, Tainan, Taiwan

**Keywords:** Signal peptide, Deep learning, Semantic segmentation

## Abstract

**Background:**

Signal peptides play an important role in protein sorting, which is the mechanism whereby proteins are transported to their destination. Recognition of signal peptides is an important first step in determining the active locations and functions of proteins. Many computational methods have been proposed to facilitate signal peptide recognition. In recent years, the development of deep learning methods has seen significant advances in many research fields. However, most existing models for signal peptide recognition use one-hidden-layer neural networks or hidden Markov models, which are relatively simple in comparison with the deep neural networks that are used in other fields.

**Results:**

This study proposes a convolutional neural network without fully connected layers, which is an important network improvement in computer vision. The proposed network is more complex in comparison with current signal peptide predictors. The experimental results show that the proposed network outperforms current signal peptide predictors on eukaryotic data. This study also demonstrates how model reduction and data augmentation helps the proposed network to predict bacterial data.

**Conclusions:**

The study makes three contributions to this subject: (a) an accurate signal peptide recognizer is developed, (b) the potential to leverage advanced networks from other fields is demonstrated and (c) important modifications are proposed while adopting complex networks on signal peptide recognition.

## Background

Protein sorting is the mechanism whereby proteins are transported to their destination inside and/or outside cells. Signal peptides play an important role in this process [1]. Proteins with signal peptides enter the secretory pathway and are then be transported to appropriate organelles, where the proteins fulfill their functions. Signal peptides operate as a permission gateway for the transport of proteins into the endoplasmic reticulum. Blobel and Sabatini [2] observed an interaction between ribosome and endoplasmic reticulum in 1971. In 1972, Milstein et al. [3] proposed that an extra sequence fragment might exist at the N-terminus of a polypeptide, which serves as a signal transmitter for the translocation of proteins. In 1975, Blobel and Dobberstein [4, 5] proposed a signal hypothesis that believed the signal sequence is located at the N-terminus of a polypeptide and is downgraded after protein translocation.

The term “signal peptide” was first coined in a study by von Heijne [1], which defined some basic properties of signal peptides. The study found that signal peptides are short amino acid sequences that are located at the N-terminus of proteins. The length of a signal peptide ranges from 11 to 27 residues. From the N-terminus, a signal peptide is composed of three sections. The first section is a positively charged n-region with about 1~5 residues. The second section is a hydrophobic h-region with about 7~15 residues. The final section is a polar uncharged c-region with about 3~7 residues. The end of signal peptides is called cleavage site.

The recognition of signal peptides is an important first step in determining the active locations and functions of proteins [6]. An effective method of determining signal peptide sequences is to read the sequences of a newborn protein and the corresponding mature protein via in vitro experiments. However, these in vitro experiments are considerably costly. Therefore, many computational methods have been proposed to facilitate signal peptide recognition. The first computational method for signal peptide recognition was proposed in 1983. Von Heijen proposed a statistical method based on 78 eukaryotic proteins [7]. A (− 3, − 1)-rule was proposed, which refers to a specific pattern at the first and the third positions before the cleavage site. In 1986, the same research group proposed an algorithm that uses a weight matrix to recognize signal peptides [8]. In 1998, Nielsen and Krogh used a hidden Markov model (HMM) to fit the three section-property and (− 3, − 1)-rule of signal peptides [9]. In 1997, Nielsen et al. proposed a method that uses a neural network (NN) and achieved much better performance than other contemporary methods [10]. In 2004, Bendtsen et al. proposed the SignalP 3.0 algorithm, which combines HMM and NN [11]. In 2011, the same research group proposed the SignalP 4.0 algorithm, which combines two neural networks [12]. The SignalP 4.0 algorithm has become a paradigm in the field of signal peptide recognition. The study also showed that many methods produce high false-positive rates for misclassified proteins that treat transmembrane helices as signal peptides.

In recent years, the development of deep learning methods has seen significant advances in many research fields. Specifically, convolutional neural networks (CNN) [13] have been used to achieve excellent performance in image classification [14, 15]. Recurrent neural networks (RNN) [16] have been used for time series data [17]. In addition, the networks have been used with great success in the field of molecular biology [18, 19]. In 2017, Savojardo et al. proposed the DeepSig algorithm [6], which is the first CNN-based method that predicts whether an amino acid sequence contains signal peptides.

This study proposes a CNN architecture without fully connected layers for signal peptide recognition. Neural networks without fully connected layers have been widely used in semantic segmentation of images with great success. For example, the fully convolutional network (FCN) [20], U-Net [21] and DeepLab [22] are three CNN architectures that are designed for semantic segmentation of images. This study modifies U-Net to process protein sequences. The modified network, named SigUNet in the context, is different to U-Net in that it (a) processes one-dimensional data, (b) adjusts the down-sampling strategy to prevent the loss of information, (c) reduces model complexity for small datasets and (d) is a trainable network architecture. The experimental results in this study show that SigUNet outperforms current signal peptide predictors on eukaryotic data. This study also demonstrates how model reduction and data augmentation helps the proposed network to predict bacterial data.

## Results

### Experimental design

Similar to previous studies [6, 12], Matthews Correlation Coefficient (MCC) and the false-positive rate for transmembrane proteins (*FPR*_*TM*_) are two main evaluation indices adopted in this study. MCC measures the correlation between the observed and predicted classes. *FPR*_*TM*_ measures the probability that a transmembrane protein is misclassified as a signal peptide. Signal peptides and N-terminal transmembrane helices are highly similar, except that transmembrane helices usually have longer hydrophobic regions and have no cleavage sites. *FPR*_*TM*_ is used to measure the ability to discriminate signal peptides from transmembrane proteins. This study also uses precision, recall and F1 measure as supplemental indices. Precision measures the fraction of real signal peptides in samples that are predicted to be signal peptides. Recall measures the fraction of signal peptides that are correctly predicted to be signal peptides. F1 measure is the harmonic mean of precision and recall. The three indices are widely used in binary classification. The details of these evaluation indices are described in the Materials and Methods section.

Table 1 shows the datasets that are used to evaluate signal peptide recognition. The details of how the datasets are constructed are in the Materials and Methods section. The SignalP dataset was constructed in 2011 by Petersen et al. [12] and the SPDS17 dataset was constructed in 2017 by Savojardo et al. [6]. Petersen et al. defined a subset of the SignalP dataset as a comparison dataset. Savojardo et al. constructed the SPDS17 dataset as another comparison dataset to accommodate newly discovered proteins. Both datasets are separated into Eukaryotes, Gram-positive bacteria and Gram-negative bacteria subsets because Hejine showed that signal peptides in different groups of organisms have different lengths and amino acid compositions [1]. Pertersen el al. and Savojardo et al. adopted a nested cross validation procedure to evaluate their methods. The procedure uses an inner cross validation to prevent peeking at the comparison dataset while the hyper-parameters are tuned. This study uses the same evaluation procedure. The details of the dataset construction and the nested cross validation are described in the Materials and Methods section.

**Table 1:** Statistics of the datasets that are used in this study

Organism	Signal Peptides		Transmembrane		Cytosolic or Nuclear		Total
	Train	Comp	Train	Comp	Train	Comp	
SignalP							
Eukaryotes	1640	606	987	939	5133	1000	7760
Gram-positive	208	48	117	117	360	213	685
Gram-negative	423	104	523	523	912	260	1858
SPDS17							
Eukaryotes	–	46	–	323	–	689	1058
Gram-positive	–	9	–	189	–	240	438
Gram-negative	–	23	–	89	–	99	211

The SignalP dataset is from the UniProtKB/Swiss-Prot in accordance with the identity list in Pertersen et al.’s study [12]; The SPDS17 dataset is from the UniProtKB/Swiss-Prot in accordance with the identity list in Savojardo et al.’s study [6].

### The performance on the eukaryotes datasets

Table 2 compares the results of ten alternative methods and SigUNet on the Eukaryotes dataset. Of the 11 methods, DeepSig and SigUNet use deep neural networks. The other nine methods use one-hidden-layer NN or HMM models and SignalP 4.0 is the most accurate of them. SigUNet outperforms the other models in terms of both MCC and *FPR*_*TM*_. For the SignalP dataset, DeepSig achieves a comparable MCC and a better *FPR*_*TM*_ than SignalP 4.0. SigUNet gives a similar *FPR*_*TM*_ and a 3.0% better MCC than DeepSig. The 4.3% gap in recall between SigUNet and DeepSig shows that SigUNet captures more signal peptides. For the SPDS17 dataset, DeepSig outperforms SignalP 4.0 in terms of both MCC and *FPR*_*TM*_. SigUNet gives a 3.5% better MCC than DeepSig. Unlike the SignalP dataset, this improvement is due to a low *FPR*_*TM*_ and not a high recall. Namely, SigUNet discriminates more transmembrane proteins from signal peptides on the SPDS17 dataset. These results show that SigUNet performs well on eukaryotic signal peptides, regardless of the dataset that is used.

**Table 2:** The performance on the Eukaryotes datasets

Method	MCC (%)	*FPR*_*TM*_ (%)	Precision (%)	Recall (%)	F1 measure (%)
The SignalP dataset					
Phobius	81.1	15.3	77.6	95.2	85.5
PrediSi	56.1	52.6	52.0	91.3	66.3
SignalP3.0-HMM	75.9	23.5	69.5	97.4	81.1
SignalP3.0-NN	56.2	64.1	48.4	**98.8**	65.0
PolyPhobius	80.6	12.5	79.5	91.9	85.2
Philius	80.4	13.4	77.8	93.7	85.0
SPOCTOPUS	80.1	14.0	79.0	91.7	84.9
SignalP 4.0	87.4	6.1	–	–	–
TOPCONS2	84.6	9.6	83.6	93.6	88.3
DeepSig	87.2	4.2	92.5	87.8	90.1
SigUNet	**90.2**	**4.0**	**93.0**	92.1	**92.5**
The SPDS17 dataset					
Phobius	65.8	9.6	47.8	**95.7**	63.8
PrediSi	38.5	43.3	20.7	89.1	33.6
SignalP3.0-HMM	51.6	22.3	31.2	**95.7**	47.1
SignalP3.0-NN	36.0	59.1	17.5	**95.7**	29.5
PolyPhobius	72.0	8.0	56.4	**95.7**	71.0
Philius	62.3	6.5	44.3	93.5	60.1
SPOCTOPUS	54.0	16.4	37.9	84.8	52.3
SignalP 4.0	81.9	4.0	75.0	91.3	82.3
TOPCONS2	73.9	5.6	60.6	93.5	73.5
DeepSig	86.1	2.5	82.4	91.3	86.6
SigUNet	**89.6**	**1.2**	**91.1**	89.1	**90.1**

The performances of Phoibus, PrediSi and SignalP 3.0 are obtained from their online services (Phobius: http://phobius.sbc.su.se/; PrediSi: http://www.predisi.de/predisi/; SignalP 3.0: http://www.cbs.dtu.dk/services/SignalP-3.0/) [11, 23, 24]. The performances of PolyPhobius, Philius, SPOCTOPUS and TOPCONS2 are obtained from the TOPCONS2 software (https://github.com/ElofssonLab/TOPCONS2) [25–28]. The performance of SignalP 4.0 on the SignalP dataset is obtained from the original paper [12] and the performance on the SPDS17 dataset is obtained from its online service (http://www.cbs.dtu.dk/services/SignalP-4.0/). The performance of DeepSig on the SignalP dataset is obtained by reproducing the algorithm and the performance on the SPDS17 dataset is obtained using the source code (https://github.com/BolognaBiocomp/deepsig). For each dataset, the best performance is highlighted in bold.

### The performance on the bacteria datasets

Table 3 shows the results on the Gram-positive datasets. The performance of SignalP 4.0, DeepSig and SigUNet shows no consistent order on the SignalP and SPDS17 datasets. DeepSig gives the worst MCC on the SignalP dataset but the best MCC on the SPDS17 dataset. The results on the Gram-negative datasets show a similar phenomenon (Table 4). SignalP 4.0 gives the best MCC on the SignalP dataset but the worst MCC on the SPDS17 dataset. As a result, Tables 3 and 4 show that SigUNet does not achieve a dominant performance as it shows in Table 2. In comparison with the Eukaryotes datasets, the bacteria datasets are smaller. The SignalP Gram-positive dataset possesses 685 samples, which is merely 8.8% in comparison with the 7760 samples of the SignalP Eukaryotes dataset. It is speculated that the small size of the bacterial datasets affects the performance of SigUNet. The next section discusses the size issue in more detail.

**Table 3:** The performance on the Gram-positive datasets

Method	MCC (%)	*FPR*_*TM*_ (%)	Precision (%)	Recall (%)	F1 measure (%)
The SignalP dataset					
Phobius	67.7	20.5	60.0	87.5	71.2
PrediSi	40.9	54.7	35.0	75.0	47.7
SignalP3.0-HMM	55.8	43.6	44.3	89.6	59.3
SignalP3.0-NN	47.2	56.4	34.9	**91.7**	50.6
PolyPhobius	71.1	16.2	66.1	85.4	74.5
Philius	69.6	15.4	64.1	85.4	73.2
SPOCTOPUS	73.9	15.4	67.2	89.6	76.8
SignalP 4.0	**85.1**	**2.6**	–	–	–
TOPCONS2	81.6	6.8	80.8	87.5	**84.0**
DeepSig	73.9	6.8	81.4	72.9	76.9
SigUNet	76.1	5.1	**85.4**	72.9	78.7
The SPDS17 dataset					
Phobius	35.0	13.6	17.9	**77.8**	29.2
PrediSi	14.3	64.0	5.0	**77.8**	9.5
SignalP3.0-HMM	27.3	27.0	11.9	**77.8**	20.6
SignalP3.0-NN	16.1	45.5	5.7	**77.8**	10.7
PolyPhobius	34.5	13.2	17.5	**77.8**	28.6
Philius	30.3	79.0	16.2	66.7	26.1
SPOCTOPUS	30.3	13.8	16.2	66.7	26.1
SignalP 4.0	50.3	**0.0**	40.0	66.7	50.0
TOPCONS2	38.1	4.2	24.0	66.7	35.3
DeepSig	**54.5**	0.1	**46.2**	66.7	**54.4**
SigUNet	40.9	2.1	40.0	44.4	42.1

The best performance is highlighted in bold

**Table 4:** The performance on the Gram-negative datasets

Method	MCC (%)	*FPR*_*TM*_ (%)	Precision (%)	Recall (%)	F1 measure (%)
The SignalP dataset					
Phobius	59.9	22.6	43.9	94.2	59.9
PrediSi	30.6	69.0	19.7	86.5	32.1
SignalP3.0-HMM	47.7	39.2	31.6	93.3	47.2
SignalP3.0-NN	36.7	61.0	22.1	**95.2**	35.9
PolyPhobius	60.7	21.4	45.0	94.2	60.9
Philius	65.9	14.9	51.3	94.2	66.4
SPOCTOPUS	64.7	17.0	50.8	92.3	65.5
SignalP 4.0	**84.8**	**1.5**	–	–	–
TOPCONS2	70.8	13.2	57.2	**95.2**	71.5
DeepSig	81.2	1.7	**88.9**	76.9	**82.5**
SigUNet	80.6	**1.5**	88.8	76.0	81.9
The SPDS17 dataset					
Phobius	69.5	18.0	56.4	95.7	71.0
PrediSi	35.4	66.3	25.0	87.0	38.8
SignalP3.0-HMM	65.4	21.3	51.2	95.7	66.7
SignalP3.0-NN	49.1	44.9	33.8	95.7	50.0
PolyPhobius	75.9	13.5	62.2	100.0	76.7
Philius	88.7	2.2	84.6	95.7	89.8
SPOCTOPUS	62.5	20.2	50.0	91.3	64.6
SignalP 4.0	92.5	**0.0**	**100.0**	87.0	93.0
TOPCONS2	85.9	5.6	76.7	**100.0**	86.8
DeepSig	95.0	**0.0**	**100.0**	91.3	95.5
SigUNet	**97.6**	1.1	95.8	**100.0**	**97.9**

The best performance is highlighted in bold

### Model reduction and data augmentation

The SignalP 4.0 model has only one hidden layer and less than 20,000 trainable weights. The DeepSig model uses convolutional layers and has 20,000~100,000 trainable weights. SigUNet has 100,000~300,000 trainable weights which is three to five folds more than that of DeepSig. This study conducts two experiments to explore whether (a) model reduction and (b) data augmentation improves the performance of SigUNet on the bacteria datasets. For the first experiment, a reduced version of SigUNet, named SigUNet-light, is implemented. The number of trainable weights of SigUNet-light is reduced to 60,000~200,000. The model details are described in the Materials and Methods section. The reduced version gives a 0.8~2.3% increase in the MCC over SigUNet on the bacteria datasets, but the same effect is not observed on the SPDS17 Gram-negative dataset (Table 5). The reduced version gives a worse performance than SigUNet on the Eukaryotes datasets. This reveals that the Eukaryotes data is sufficient to train SigUNet and no model reduction is required.

**Table 5:** The performance of model reduction

Method	Eukaryotes		Gram-positive		Gram-negative	
	MCC (%)	*FPR*_*TM*_ (%)	MCC (%)	*FPR*_*TM*_ (%)	MCC (%)	*FPR*_*TM*_ (%)
The SignalP dataset						
SigUNet	90.2	4.0	76.1	5.1	80.6	1.5
SigUNet-light	89.4	4.3	**77.7**	5.1	**82.9**	1.9
The SPDS17 dataset						
SigUNet	89.6	1.2	40.9	2.1	97.6	1.1
SigUNet-light	84.8	3.7	**51.7**	**1.6**	92.8	1.1

Performances that are improved after model reduction are highlighted in bold.

For the second experiment, training data from different organisms is merged to construct larger training sets (Table 6 and Table 7). For the Eukaryotes datasets in both tables, the best MCC is achieved by training SigUNet using only the Eukaryotes data. This echoes that the Eukaryotes data is sufficient to train SigUNet. Adding bacteria data to the training set introduces noises, which mitigate the benefit of data augmentation.

**Table 6:** The performance of data augmentation on the SignalP dataset

Comp	Eukaryotes		Gram-positive		Gram-negative	
Train	MCC (%)	*FPR*_*TM*_ (%)	MCC (%)	*FPR*_*TM*_ (%)	MCC (%)	*FPR*_*TM*_ (%)
SigUNet						
As comp^a^	**90.2**	4.0	76.1	5.1	80.6	1.5
All organisms^b^	89.9	**3.2**	80.9	3.1	82.1	3.6
Bacteria^c^	–	–	79.3	**1.9**	83.5	**0.3**
SigUNet-light						
As comp	89.4	4.3	77.7	5.1	82.9	1.9
All organisms	88.9	3.9	**82.5**	3.1	81.4	3.5
Bacteria	–	–	80.2	**1.9**	**83.9**	2.7

^a^The model is trained using the same organism as the comparison dataset. ^b^The model is trained using all organisms. ^c^The model is trained using all of the bacteria data. The best performance is highlighted in bold

**Table 7:** The performance of data augmentation on the SPDS17 dataset

Comp	Eukaryotes		Gram-positive		Gram-negative	
Train	MCC (%)	*FPR*_*TM*_ (%)	MCC (%)	*FPR*_*TM*_ (%)	MCC (%)	*FPR*_*TM*_ (%)
SigUNet						
As comp^a^	**89.6**	**1.2**	40.9	2.1	97.6	1.1
All organisms^b^	89.2	2.2	46.1	1.6	**100.0**	**0.0**
Bacteria^c^	–	–	49.5	**1.1**	97.6	1.1
SigUNet-light						
As comp	84.8	3.7	**51.7**	1.6	92.8	1.1
All organisms	89.1	2.2	43.3	1.6	**100.0**	**0.0**
Bacteria	–	–	49.5	**1.1**	**100.0**	**0.0**

^a^The model is trained using the same organism as the comparison dataset. ^b^The model is trained using all organisms. ^c^The model is trained using all of the bacteria data. The best performance is highlighted in bold

If training involves all organisms, the *FPR*_*TM*_ is improved in three of the four scenarios (SigUNet and SigUNet-light on the SignalP dataset and SigUNet-light on the SPDS17 dataset). A better *FPR*_*TM*_ indicates that more transmembrane proteins are discriminated from signal peptides. This suggests that the properties of transmembrane proteins are less different to those of signal peptides across organisms. On the Gram-positive datasets, The best *FPR*_*TM*_ is achieved using bacteria data for training. This suggests that some Gram-positive transmembrane proteins are similar to eukaryotic signal peptides, which decreases the ability to discriminate Gram-positive transmembrane proteins from signal peptides. On the Gram-negative datasets, both data augmentation strategies work. Training with bacterial data gives the best MCC and *FPR*_*TM*_ on the SignalP Gram-negative dataset; while training with all organisms gives the best MCC and *FPR*_*TM*_ on the SPDS17 Gram-negative dataset. These results reveal that data augmentation improves the performance of SigUNet on the bacterial datasets.

In summary, SigUNet is suited to the recognition of eukaryotic signal peptides. Its network architecture requires a relatively large dataset for training. Model reduction and data augmentation are useful, but increasing the amount of data is still required to ensure that SigUNet recognizes bacterial signal peptides.

## Discussion

The Results section compares the performance of the methods and demonstrates the issues of SigUNet in terms of data size. This section discusses the variation in performance by analyzing the sequence composition. Training speed, which is highly dependent on data size, is also discussed in this section.

**Fig. 1:**
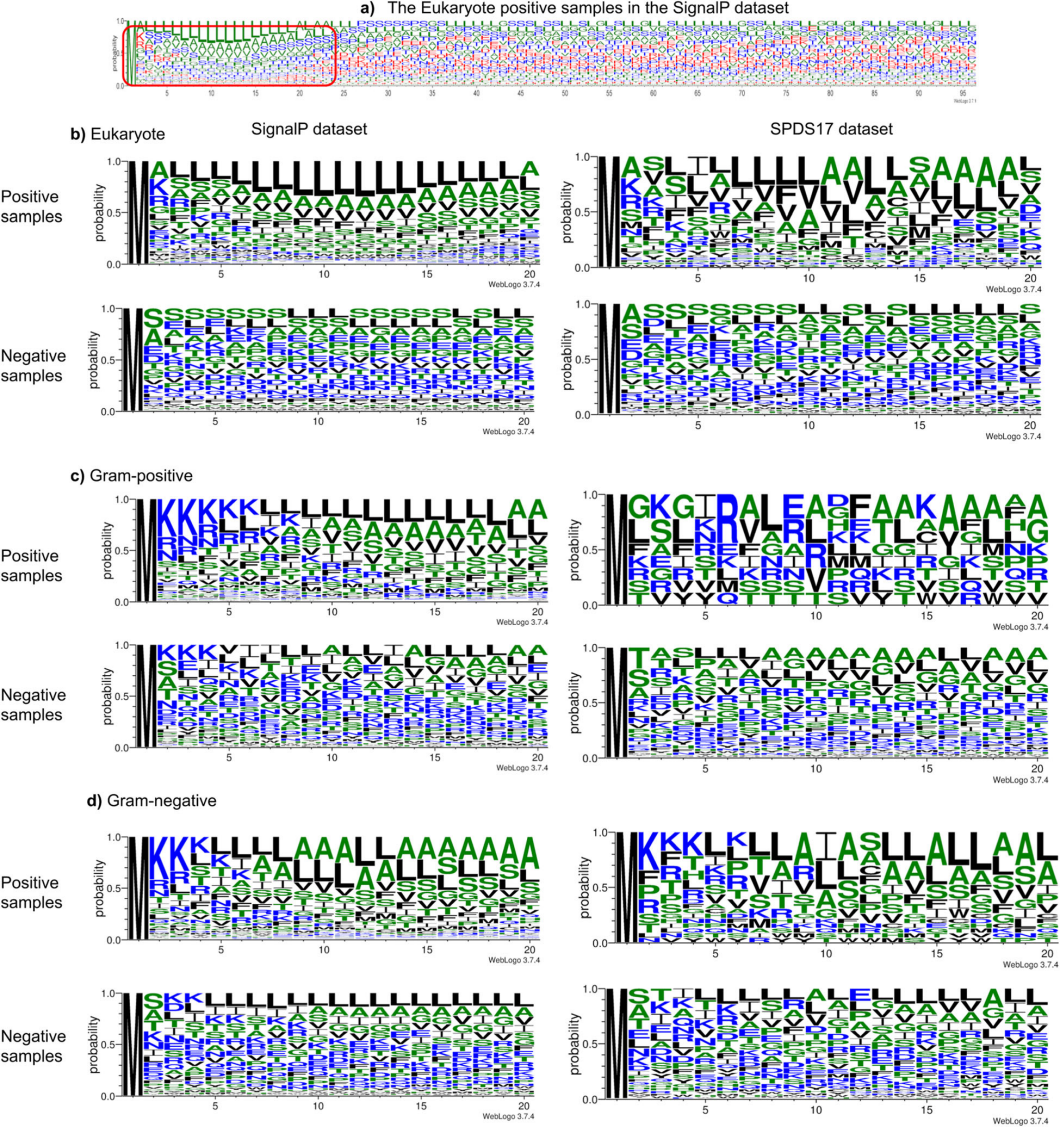
Sequence logos generated by WebLogo [29]. The *x*-axis indicates the position of the amino acid and the *y*-axis shows the probabilities of amino acids across a given sequence set. **a** Sequence logo for 96 positions for the SignalP Eukaryotes dataset. **b** Sequence logos for the first 20 positions for the Eukaryotes datasets. **c** Sequence logos for the first 20 positions for the Gram-positive datasets. **d** Sequence logos for the first 20 positions for the Gram-negative datasets. Non-polar, charged and polar amino acids are respectively colored green, red and blue

To analyze the sequence composition, the sequences of each dataset are plotted into sequence logos as shown in Fig. 1. The sequence logo for 96 positions in Fig. 1a is too confusing to analyze, so the first 20 positions of each dataset are shown in Fig. 1b, c and d for clarity. The top left subplot of Fig. 1b, c and d are sequence logos plotted for the signal peptides in the SignalP datasets. Although the sequences are from different organisms, the three subplots exhibit a similar pattern. The pattern begins with a fixed M in position one followed by charged (red) amino acids and then by non-polar (green) amino acids. This is consistent with the current knowledge that signal peptides comprise a charged n-region, a hydrophobic h-region and a polar c-region.

The sequence logos of SPDS17 show a larger variation than those of SignalP across organisms. The top right subplot of Fig. 1c is more random than other sequence logos that are plotted for signal peptides. This explains why no method gives satisfactory results on the SPDS17 Gram-positive data. Conversely, both of the top left and top right subplots of Figure 1d have three obvious ‘K’s in positions 2, 3 and 4. This explains why SigUNet and other methods perform well on the SPDS17 Gram-negative data.

**Fig. 2:**
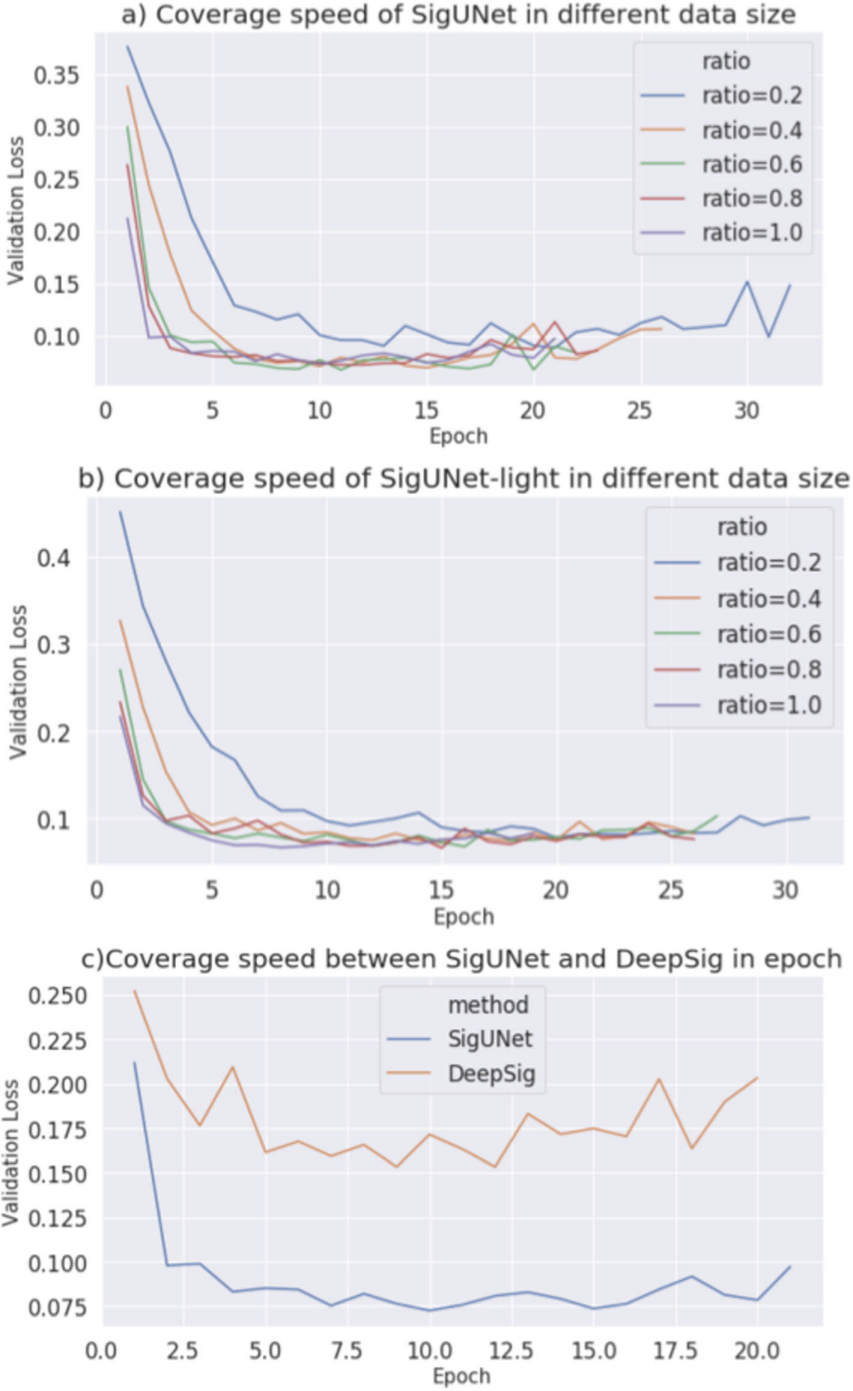
Epoch-loss plots of training SigUNet. **a** Training SigUNet using different ratios of SignalP Eukaryotes data. **b** Training SigUNet-light using different ratios of SignalP Eukaryotes data. **c** Training DeepSig and SigUNet using the SignalP Eukaryotes data

To analyze the training speed, SigUNet was trained using datasets of different sizes. Figure 2 shows the epoch-loss plots. Figure 2a shows that SigUNet stops after a similar number of epochs when 100, 80 and 60% of the data is used. As the time that is required to train an epoch is proportional to the size of the dataset, the training time for SigUNet is linearly proportional to the size of the dataset. The validation losses of the three lines are similar, which shows that 60% of Eukaryotes data is sufficient to train SigUNet. When only 40% or 20% of the data is used, the validation loss is bumpy and SigUNet requires more epochs to train. SigUNet-light gives a similar result. Figure 2b shows that SigUNet-light stops after a similar number of epochs when 100, 80, 60 and 40% of the data is used. Namely, 40% of the Eukaryotes data is sufficient to train the reduced version of SigUNet.

Figure 2c compares the training speed of SigUNet with that for DeepSig. DeepSig stops earlier than SigUNet, but SigUNet gives a lower validation loss. SigUNet is more complex than DeepSig, so these observations are consistent with the common knowledge that simpler models converge faster but perform worse. An interesting observation is that the validation loss of DeepSig is bumpier than that of SigUNet. This shows that SigUNet has more stable training process than DeepSig. In addition to network architecture, there is an obvious difference between DeepSig and SigUNnet in terms of the loss function. The loss function of DeepSig calculates the protein-level cross entropy and SigUNet calculates the amino acid-level cross entropy. Figure 2c shows that the gradient that is generated by the loss function of SigUNet updates the model more smoothly. This observation is pertinent to future signal peptide studies for the development of loss functions.

## Conclusions

This study proposes a new deep learning model for signal peptide recognition. The proposed model is more complex than those of previous studies by leveraging network improvements that have been developed in computer vision. This study also proposes network modifications to enhance the performance on protein data. The experimental results show that the proposed model outperforms conventional neural networks. This conclusion is consistent with SignalP 5.0 [30], which was published on 18 February 2019. Although SignalP 5.0 uses a different evaluation procedure, it gives similar results when advanced network architectures are used.

## Materials and methods

### Evaluation indices

This work uses the Matthews Correlation Coefficient (MCC) to evaluate signal peptide recognition. The MCC measures the correlation between two series of binary data. In practice, the MCC is usually used as an overall index for binary classification by establishing the observed classes as one data series and the predicted classes as the other data series. The MCC is shown as below:

- The definition of the Matthews Correlation Coefficient

1 $$ \mathrm{MCC}=\frac{TP\times TN- FP\times FN}{\sqrt{\left( TP+ FP\right)\times \left( TP+ FN\right)\times \left( TN+ FP\right)\times \left( TN+ FN\right)}} $$

In Eq. 1, TP indicates true positive, which is the number of signal peptides that are correctly predicted to be signal peptides; TN indicates true negative, which is the number of non-signal peptides that are correctly predicted to be non-signal peptides; FP indicates false positive, which is the number of non-signal peptides that are incorrectly predicted to be signal peptides; and FN indicates false negative, which is the number of signal peptides that are incorrectly predicted to be non-signal peptides. The characteristics of signal peptides and N-terminal transmembrane helices are similar, so signal peptide predictors must be able to discriminate signal peptides from transmembrane proteins. This study uses the false positive rate for transmembrane proteins (*FPR*_*TM*_) to measure this ability:

- The definition of the false positive rate for transmembrane proteins

2 $$ { FP R}_{TM}=\frac{FP_{TM}}{N_{TM}} $$

In Equation 2, *N*_*TM*_ represents the total quantity of transmembrane proteins and *FP*_*TM*_ represents the number of transmembrane proteins that are misclassified as signal peptides. MCC and *FPR*_*TM*_ are the main evaluation indices adopted in SignalP 4.0 and DeepSig. This study also uses precision, recall and F1 measure, which are widely used evaluation indices for binary classification:

- The definition of precision

3 $$ Precision=\frac{TP}{TP+ FP} $$

- The definition of recall

4 $$ Recall=\frac{TP}{TP+ FN} $$

- The definition of F1 measure

5 $$ F1=\frac{2\times Precision\times Recall}{Precision+ Recall}=\frac{2\times TP}{2\times TP+ FN+ FP} $$

Precision measures the ratio of correctness when a protein is reported to be a signal peptide; recall measures the fraction of signal peptides that are correctly captured. Precision is an index of exactness or quality and recall is an index of completeness or quantity. F1 measure, which is the harmonic mean of precision and recall, is commonly optimized to balance precision and recall.

### Datasets

Two datasets are used in this study: the SignalP and SPDS17 datasets (Table 1). The SignalP dataset contains three subsets: Eukaryotes, Gram-positive and Gram-negative bacteria. It uses proteins from the UniProtKB/Swiss-Prot release 2010_05 [31] and excludes hypothetical proteins and proteins with less than 30 amino acids. Positive samples in the SignalP dataset are signal peptides with experimentally verified cleavage sites. Negative samples are (a) proteins whose subcellular locations are only nuclear or cytosolic and (b) proteins whose first 70 amino acids are tagged as a transmembrane region. A homology reduction algorithm that was proposed by Hobohm et al. [32] is applied to the first 70 amino acids. This algorithm considers two proteins for which the local alignment has more than 17 identical amino acids as redundant for Eukaryotes and two proteins for which the local alignment has more than 21 identical amino acids as redundant for bacteria. A small part of the SignalP dataset was used as a comparison dataset by Petersen et al. [12].

The SPDS17 dataset was constructed by Savojardo et al. [6]. It contains proteins from UniProtKB/Swiss-Prot releases 2015_06 to 2017_04. Similar to the SignalP dataset, the SPDS17 dataset separates proteins into three subsets: Eukaryotes, Gram-positive bacteria and Gram-negative bacteria. The definitions of positive and negative samples are identical to those in the SignalP dataset. Namely, the SPDS17 dataset is a comparison dataset for the SignalP dataset that accommodates newly discovered proteins. The homology of the SPDS17 is reduced using the blastclust algorithm with an E-value of 0.001 [33]. Proteins with greater than a 25% similarity are considered as redundant. Proteins with a similarity higher than 25% to any protein in the SignalP dataset are removed.

### Data preprocessing

Signal peptides only appear at the front of amino acid chains, so only a fixed number of amino acids from each protein sequence are used as an input. This study uses 96 as the input length, which is the same as DeepSig. The first 96 amino acids of a protein are one-hot encoded. Namely, every amino acid is encoded into a 20-dimensional binary vector, where 19 positions are zero and only the position that corresponds to the amino acid is one. An uncommon or unknown amino acid such as ‘X’ is encoded as a zero vector. To encode all proteins into a 96 × 20 matrix, zeros are padded to vectors for proteins that have less than 96 amino acids. To determine the ability to discriminate signal peptides from transmembrane proteins, this study classifies amino acids into three classes. If an amino acid is located in a signal peptide region, it is labeled ‘S’. If an amino acid is located in a transmembrane region, it is labeled ‘T’. If an amino acid is not located in a signal peptide nor a transmembrane region, it is labeled ‘N’. The class of a protein is one-hot encoded as a 96 × 3 matrix. In summary, given a protein sequence, this study encodes it into a 96 × 20 matrix as the input. The output is a 96 × 3 matrix, which includes amino acid-level predictions for the given protein sequence.

### Network architecture

**Fig. 3:**
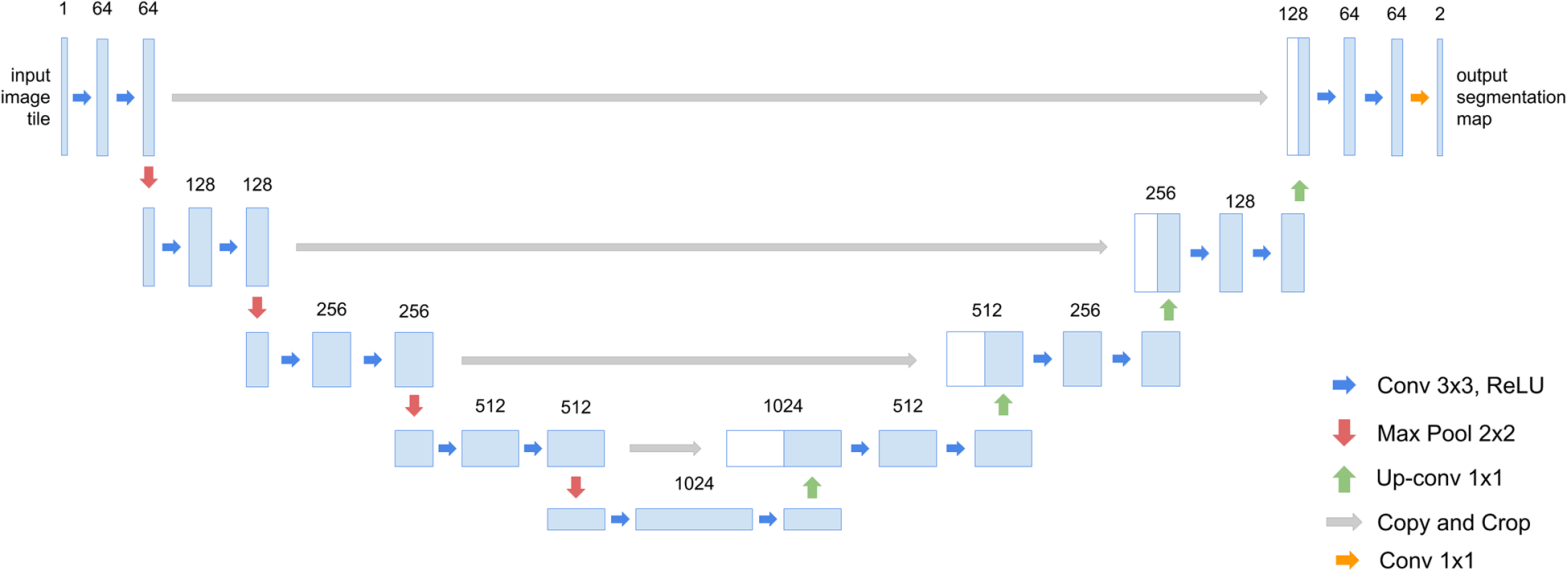
The network architecture of U-Net [21]

The network architecture of this work is based on U-Net, which achieves excellent results for the semantic segmentation of medical images [21]. Medical image datasets are much smaller than other common computer vision datasets and U-Net is tailored to this situation. Figure 3 shows the architecture of U-Net. The model input is a 572 × 572 grey scale image and the output is a 388x388x2 semantic segmented image. Convolutional layers (denoted as ‘conv 3x3 ReLU’ blue arrows and ‘conv 1 × 1’ teal arrows in Fig. 3) use filters to recognize local patterns [13]. A filter is a matrix that is convolved across the width and height of the input image to generate a feature map. The suffix (‘3x3 ReLU’ and ‘1 × 1’) indicates the size of the filter and the activation functions of the corresponding convolutional layers. The ‘copy and crop’ gray arrows in Fig. 3 copy the output of a source layer (the left end of the arrow) and crop it to fit the size of the destination layer (the right end of the arrow). Pooling layers (denoted as ‘max pool 2x2’ red arrows in Fig. 3) merge adjacent output values from previous layers into one value to reduce network complexity [34]. Max pooling uses the maximum value of a local area as the output. The suffix (‘2x2’) indicates the size of each local area that is to be merged. Up-convolutional layers (denoted as ‘up-conv 2x2’ green arrows in Fig. 3), which perform an inverse operation to convolutional layers, expand the information that is compressed by convolutional and pooling layers [35].

**Fig. 4:**
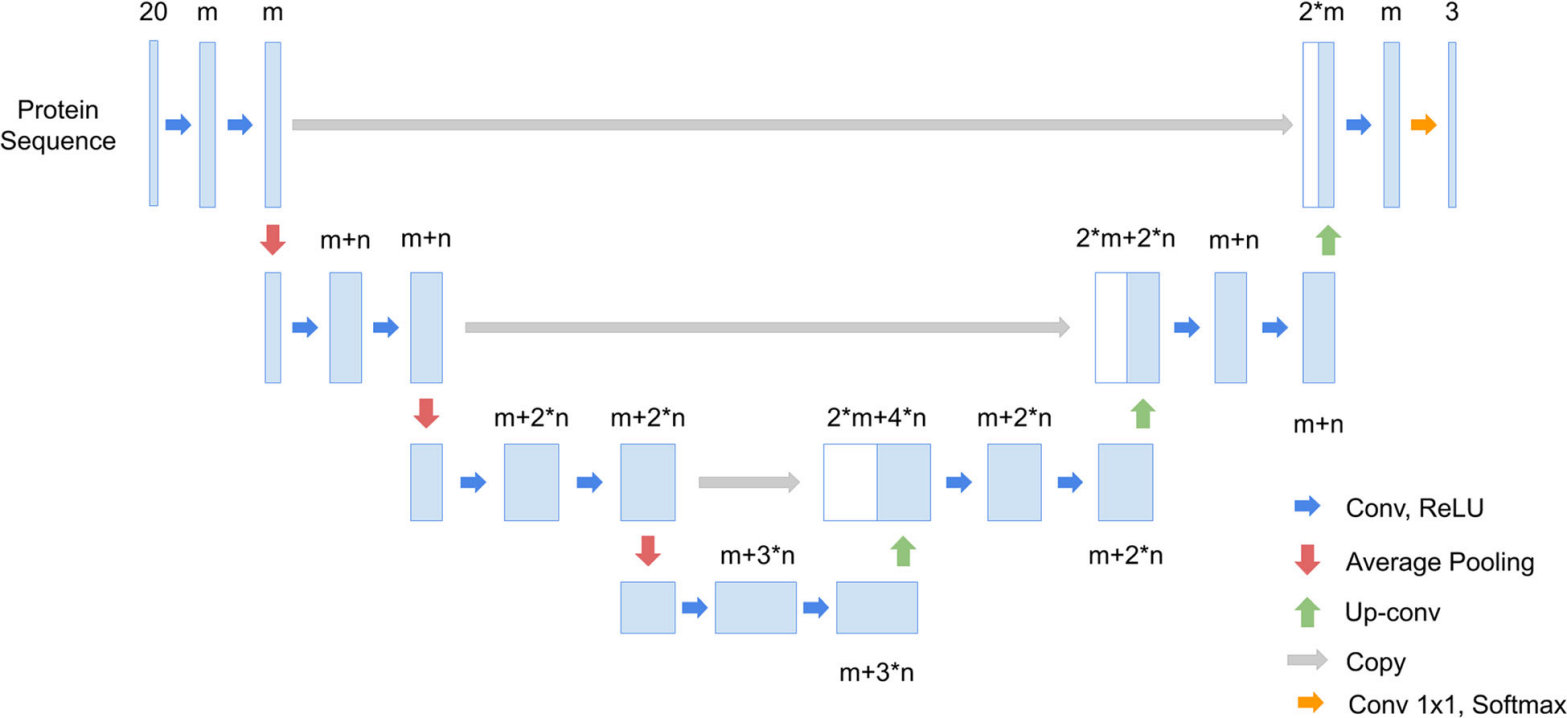
The network architecture of SigUNet

**Fig. 5:**
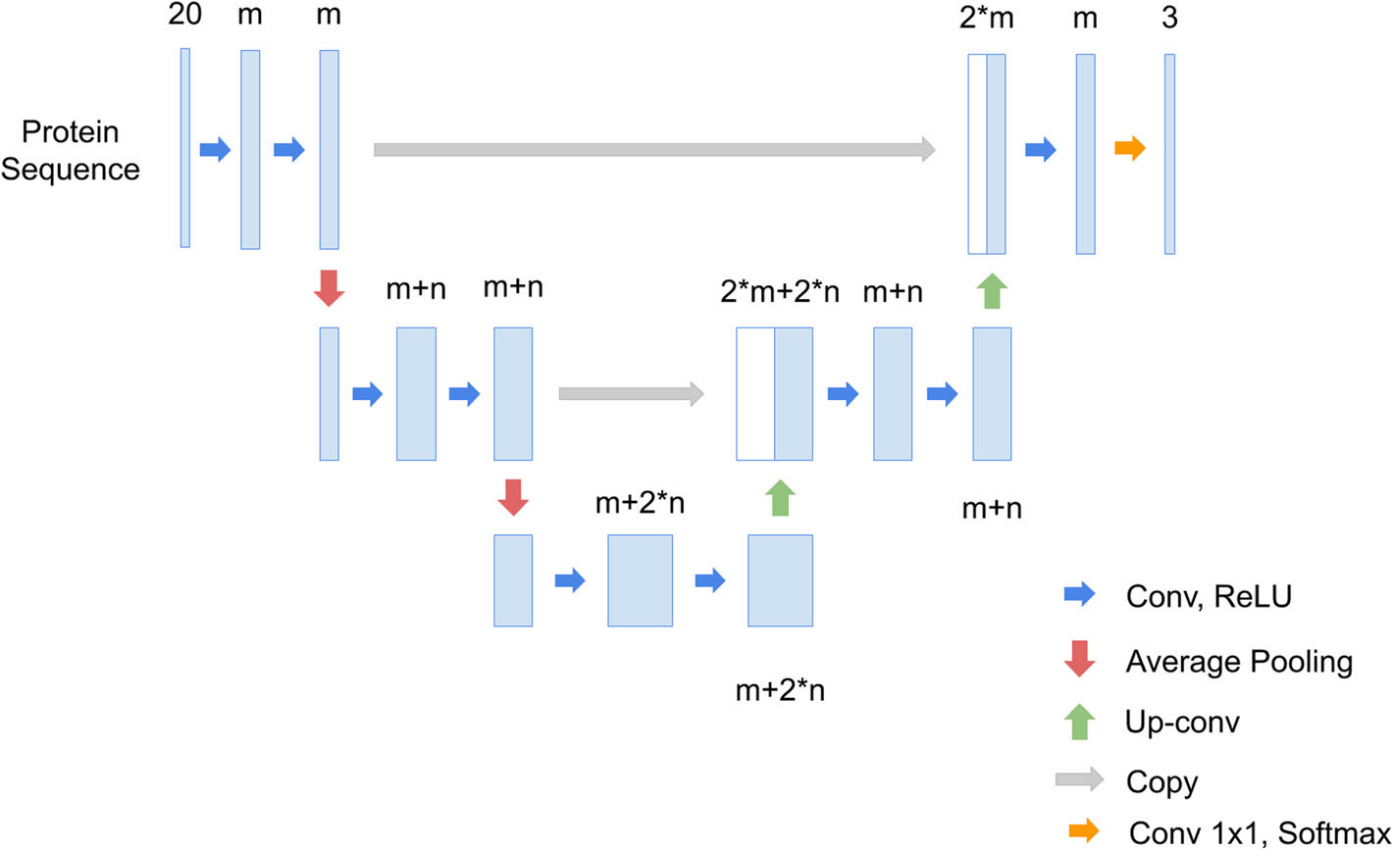
The network architecture of SigUNet-light, which is a reduced version of SigUNet

U-Net is used for two-dimensional images, so this study refines it for use with one-dimensional protein sequences. Each two-dimensional operation becomes one-dimensional and each position in a sequence is represented by a 20-channel vector. However, this trivial one-dimensional U-Net does not allow efficient signal peptide recognition (Table 8). To solve the problem, this study refines the number of channels in each layer (Fig. 4). The network architecture is named SigUNet. The original U-Net fixes the channel size of the first convolutional layer to 64 and doubles the channel size to 128, 256, 512 and 1024 after each pooling layer. This made number of parameters of U-Net increases exponentially. In SigUNet, the channel size starts from *m* and increases linearly by *n*. Both *m* and *n* are hyper-parameters that are determined using nested cross validation. Unlike pixels in an image, it is hypothesized that each amino acid contains important information and is not disposable. Using max pooling, the information in an amino acid can be lost if its neighbor has a large value. Therefore, average pooling is adopted in SigUNet. Table 8 shows the performance of using different pooling operations. A reduced version of SigUNet for bacteria signal peptides is shown in Fig. 5. The reduced SigUNet is named SigUNet-light.

**Table 8:** The performance of different network architectures on the SignalP Eukaryotes dataset

Architecture	MCC (%)	*FPR*_*TM*_ (%)	Recall (%)	Precision (%)	F1 measure (%)
U-Net-1D^a^	84.1	6.8	87.3	88.5	87.9
SigUNet-max^b^	88.6	5.0	91.4	91.3	91.3
SigUNet	90.2	4.0	92.1	93.0	92.5

^a^A one-dimensional U-Net that has the same network configuration as Fig. 3, but the input and output layer are modified for protein sequences. ^b^The max pooling layers in Fig. 4 are replaced with average pooling layers

The architecture of SigUNet outputs a 96 × 3 matrix that represents the probabilities of the 96 amino acids being classified as either a signal peptide, a transmembrane region or neither. The loss function is cross entropy shown as below:

- The loss function of SigUNet

6 $$ \mathrm{Loss}\left(\mathrm{x},\mathrm{y}\right)=-\sum \limits_{i=1}^{96}\sum \limits_{j=1}^3{y}_{ij}\mathit{\ln}\left(h{\left(\mathrm{x}\right)}_{ij}\right) $$

Here x represents an input sample, which is a 96 × 20 matrix; y represents the real class of the input sample, which is one-hot encoded to a 96 × 3 matrix; *y*_*ij*_ is a binary value that indicates whether the *i*-th amino acid is of the *j*-th class; *h*(x) represents the network output, which is a 96 × 3 matrix; and *h*(x)_*ij*_ represents the probability of the *i*-th amino being of the *j*-th class. The 96 × 3 output matrix for an input sequence is then transformed to a binary prediction. If the probability of any four consecutive amino acids being a signal peptide is greater than a threshold, the input sequence is classified to be a signal peptide. The threshold is a hyper-parameter of SigUNet and is determined using nested cross validation.

### Nested cross validation

**Fig. 6:**
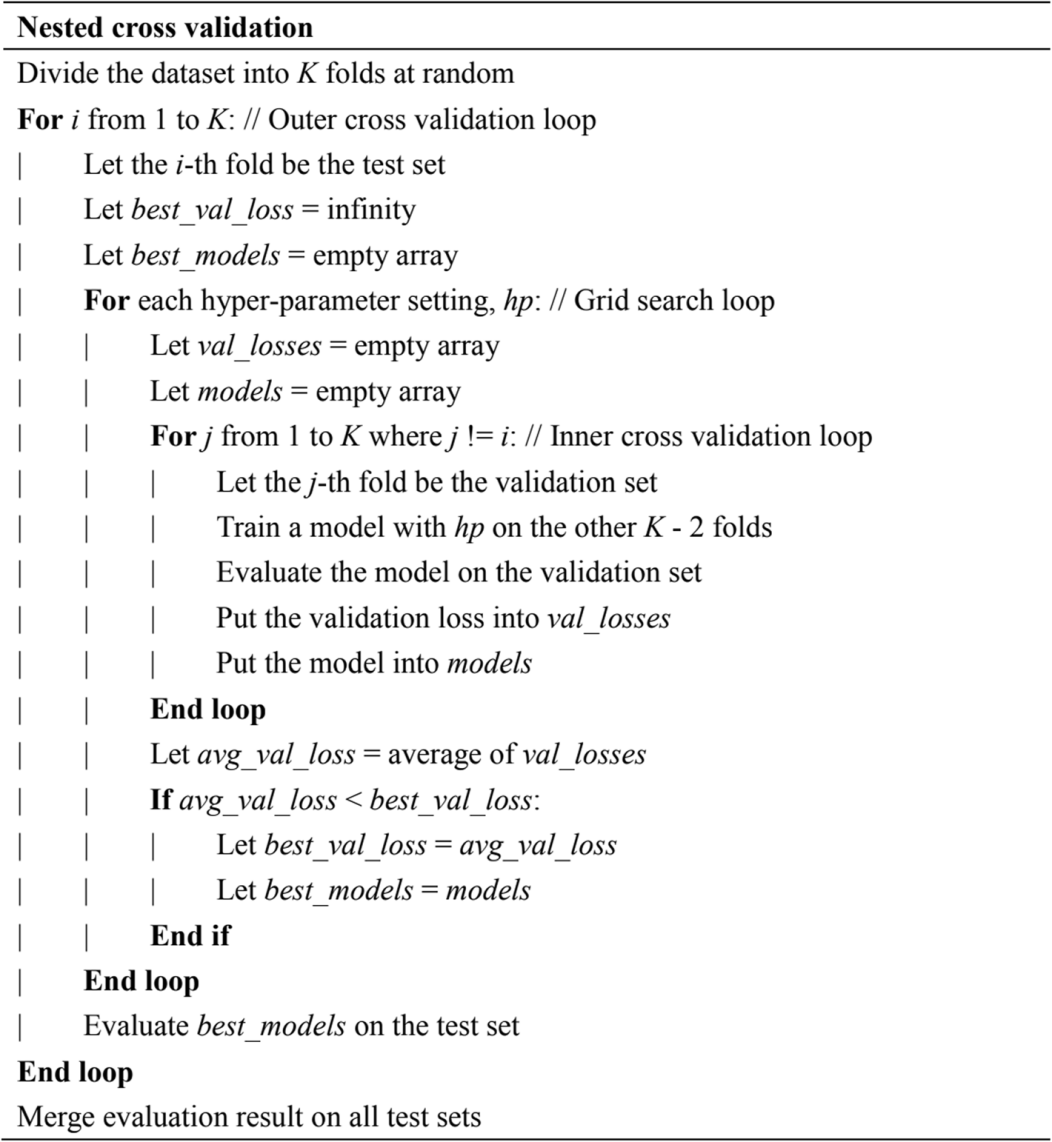
The pseudo code of nested cross validation

Cross validation is used in machine learning to prevent overfitting. For a *k*-fold cross validation, the data is split into *k* partitions. Each partition is used for testing and the remaining *k*-1 partitions are used to train a model. However, if the performance of cross validation is used to determine hyper-parameters, it is no longer an appropriate indicator for model performance. To solve this issue, this work adopts a nested cross validation procedure (Fig. 6), whereby hyper-parameters are determined using an inner *k*-1-fold cross validation on the *k*-1 training partitions. For each testing partition, the inner *k*-1-fold cross validation constructs *k*-1 models and their predictions on the testing partition are averaged. This procedure does not peek at the testing partition when the hyper-parameters are tuned. Therefore, the performance of the outer cross validation can be used to represent the model performance. The nested cross validation and *k* = 5 are the same as the evaluation procedure in SignalP 4.0 and DeepSig.

## Data Availability

The source code of SigUNet is available at https://github.com/mbilab/SigUNet.
